# Lipid Profiling Demonstrates That Suppressing *Arabidopsis* Phospholipase Dδ Retards ABA-Promoted Leaf Senescence by Attenuating Lipid Degradation

**DOI:** 10.1371/journal.pone.0065687

**Published:** 2013-06-07

**Authors:** Yanxia Jia, Faqing Tao, Weiqi Li

**Affiliations:** 1 Key Laboratory of Biodiversity and Biogeography, Kunming Institute of Botany, Chinese Academy of Science, Kunming, China; 2 The Germplasm Bank of Wild Species, Kunming Institute of Botany, Chinese Academy of Sciences, Kunming, China; 3 University of Chinese Academy of Sciences, Beijing, China; Iowa State University, United States of America

## Abstract

Senescence is the last phase of the plant life cycle and has an important role in plant development. Degradation of membrane lipids is an essential process during leaf senescence. Several studies have reported fundamental changes in membrane lipids and phospholipase D (PLD) activity as leaves senesce. Suppression of phospholipase Dα1 (PLDα1) retards abscisic acid (ABA)-promoted senescence. However, given the absence of studies that have profiled changes in the compositions of membrane lipid molecules during leaf senescence, there is no direct evidence that PLD affects lipid composition during the process. Here, we show that application of *n*-butanol, an inhibitor of PLD, and N-Acylethanolamine (NAE) 12∶0, a specific inhibitor of PLDα1, retarded ABA-promoted senescence to different extents. Furthermore, phospholipase Dδ (PLDδ) was induced in leaves treated with ABA, and suppression of PLDδ retarded ABA-promoted senescence in *Arabidopsis*. Lipid profiling revealed that detachment-induced senescence had different effects on plastidic and extraplastidic lipids. The accelerated degradation of plastidic lipids during ABA-induced senescence in wild-type plants was attenuated in PLDδ-knockout (PLDδ-KO) plants. Dramatic increases in phosphatidic acid (PA) and decreases in phosphatidylcholine (PC) during ABA-induced senescence were also suppressed in PLDδ-KO plants. Our results suggest that PLDδ-mediated hydrolysis of PC to PA plays a positive role in ABA-promoted senescence. The attenuation of PA formation resulting from suppression of PLDδ blocks the degradation of membrane lipids, which retards ABA-promoted senescence.

## Introduction

Leaf senescence, the last stage of leaf development, is an integral and important part of the plant life cycle [1], and greatly affects crop productivity [1]–[4]. For example, the delay of senescence by only a few days before harvest can improve the yields of certain forage crops significantly [5]. Leaf senescence can be induced by at least three conditions. One is natural senescence, which occurs as plants or leaves age. Another is stress-induced senescence, which results from stresses, such detachment, nutrient deficiency, darkness, and disease [1], [2]. The third is hormone-promoted senescence. Application of either ABA or ethylene to detached leaves can promote their senescence [6].

Leaf senescence is a complex process that manifests at the levels of morphology (e.g., yellowing of leaf edges), physiology (e.g., reduction of photosynthesis), cell composition (e.g., deterioration of chlorophyll and membranes), biochemistry (e.g., changes in levels of hormones), and molecular genetics (e.g., altered expression of more than 800 senescence-associated genes) [2]. Manipulation of certain genes, such as *tmr* [7], *IPT* [8], *SAG101* [9], and *YUCCA6* [10], delays senescence. Leaf senescence is a highly regulated process, in which events occur in an ordered manner. Whereas leaf yellowing and chlorophyll deterioration are early events, the degradation of nucleic acids and proteins occurs midway through the process [11], and destruction of the plasma membrane occurs at the end of the process [12]. The changes in membrane lipids that occur in senescing leaves were reported decades ago [13], and the activity of PLD, phosphatidic acid phosphatase, lipolytic acyl hydrolase, and lipoxygenase in senescence-related lipid metabolism are well documented [13]. Nonetheless, little is known about many important aspects of leaf senescence, such as the roles of lipolytic enzymes in mediating specific changes in membrane lipids and how these changes affect membrane deterioration.

Hydrolysis of membrane phospholipids by PLD produces PA and a free head group. The PLD family comprises 12 members, which are classified into six types, PLDα (3), β (2), γ (3), δ, ε, and ζ (2). The PLDα1 and PLDδ isoforms are two of the most abundant PLDs. All six types have been characterized well in *Arabidopsis* [14]. Most plant PLDs have distinctive molecular and biochemical properties that are associated with diverse cellular and physiological roles [15]. Through genetic approaches, it has been demonstrated that PLDs play important roles in stress responses, such as those to drought, cold, and salinity [16], [17]. By exploiting the specific inhibition of *n*-butanol on the PLD-mediated PA [18], it has been shown that PLDs have roles in microtubule organization [19], [20], salicylic acid signaling [21], seedling development [19], [22], and pollen tube growth [23]. By comparing the effects of *n*-butanol and N-acylethanolamines (NAEs), which are specific inhibitors of PLDα1 [24], it was proposed that different isoforms of PLD might have differential regulatory roles in seedling development and growth [22].

The hydrolytic activity of PLD has been analyzed in senescing rose petals and bean cotyledons [25], [26]. Microarray analysis showed that during natural senescence in *Arabidopsis*, levels of expression of PLDα1 and PLDδ increased 1.75- and 2.94-fold, respectively (http://www.expressionbrowser.com/). In *Arabidopsis*, application of ABA or ethylene increases PLDα1 activity, and suppression of PLDα1 retards ABA- and ethylene-promoted leaf senescence [27]. It has been proposed that PLDα1 plays a key role in lipid metabolism in senescing membranes by producing PA, which destabilizes membranes and activates other lipid-degrading enzymes, resulting in the loss of both membrane integrity and functionality of membrane-associated proteins [13], [27]. However, there is no direct evidence that PLDα1 produces PA and affects lipid composition during leaf senescence. It remains to be established whether and how any PLD isoforms apart from PLDα1 function in leaf senescence. For example, the role of PLDδ, which is one of the two most abundant PLDs [14], remains to be investigated.

Disruption of the structural and functional integrity of cellular membranes is a major factor that contributes to senescence [27]–[30]. The disruption of membranes results primarily from the remarkable metabolism of membrane lipids. Loss of membrane phospholipids was observed during natural and ethylene-induced senescence of cut carnation flowers [31], [32]. Changes in the metabolic relationships among phospholipids, and between galactolipids and phospholipids, have been reported to occur during senescence-associated lipid breakdown. Both the natural senescence of rose petals and dark-induced senescence of cabbage leaves were found to be associated with an overall decrease in levels of membrane phospholipids, with no changes in their relative abundance [5], [26]. Levels of both extraplastidic and plastidic lipids decreased during the natural senescence of tobacco leaves [33]. During dark-induced senescence of barley leaves, decreased levels of galactolipids, monogalactosyldiacylglycerol (MGDG), and digalactosyldiacylglycerol (DGDG) were accompanied by a transient accumulation of phosphatidylcholine (PC), a major phospholipid [34]. Degradation of the chloroplast membrane occurs early in the ordered series of events that occur in senescing cells [2]. It was suggested that the metabolism of plastidic lipids occurs before that of lipids in the plasma membrane [5]. However, many questions related to the lipid metabolism of senescing leaves have yet to be answered. For example, how does the profile of membrane lipids change during the process? Does hormone-promoted senescence affect lipid metabolism, and what are the mechanisms involved? Can attenuation of lipid metabolism delay leaf senescence?

In the study reported herein, we describe the alteration of ABA-promoted senescence by the application of PLD inhibitors, changes in the expression of PLDδ, and the phenotypes of *Arabidopsis* leaves in which PLDδ has been suppressed during senescence. Lipidomic analysis based on electrospray tandem mass spectrometry (ESI-MS/MS) [35], [36] enabled comparative profiling of 125 molecular species of eight membrane lipid classes in wild-type *Arabidopsis* and *Arabidopsis* plants without PLDδ before and after detachment-induced and ABA-promoted senescence. Detailed analysis of the changes in molecular lipid species revealed the functions of PLDδ in ABA-promoted senescence. We also compared PLDα1- and PLDδ-mediated formation of PA during ABA-promoted senescence. We propose a model that describes the roles of PLDα1 and PLDδ in the retardation of ABA-promoted senescence.

## Materials and Methods

### Plant Materials, Growth Conditions, and Phytohormone Treatments

Previous studies reported the generation of a PLDδ (*PLDδ*, AT4G35790)-knockout mutant (PLDδ-KO) and PLDδ-complemented plants (PLDδ-rescue, PLDδ-RC) in the background of the *Arabidopsis* Wassilewskija (WS) ecotype [37], as well as the production of a line, PLDα1-antisense (PLDα1-AS), that was derived from the *Arabidopsis* ecotype Columbia (Col) and in which PLDα1 activity was attenuated by antisense-mediated suppression [27]. *Arabidopsis* genotypes were grown hydroponically [38] in a controlled growth chamber at 23°C (day), 20°C (night), and 60% relative humidity, under 12-hr photoperiod fluorescent lighting with an intensity of 120 µmol/m^2^/s. All plants used in a given experiment were taken from a single synchronously growing population. Leaves of the same age and with an adult morphology were collected from *Arabidopsis* plants that were approximately 6 weeks old and were rinsed briefly with sterile water. The leaves were placed with their adaxial surfaces facing upwards in Petri dishes that contained either water, 50 µM solution of ABA (Sigma, A1049), ABA plus 10 µM N-Acylethanolamine (NAE) 12∶0 (kindly provided by Professor Kent Chapman) [22], ABA plus 0.03% (v/v) *n*-butanol, *sec-*butanol, or *tert-*butanol, or 50 µM ethephon (Sigma, C0143) prepared in water. The concentrations of *n*-butanol and NAE were not exactly equimolar, but their biological effects were comparable [22]. The leaves were incubated for the indicated time at approximately 23°C under a 12-hr photoperiod and light of 120 µmol/m^2^/sec [6], [27].

### Immunoblotting of PLDδ

Total protein was extracted from detached leaves incubated in water or 50 μΜ ABA in accordance with a previously described procedure [27]. Briefly, leaves were ground with a chilled mortar and pestle in three volumes of homogenization buffer (50 mM Tris-HCl, pH 7.5; 10 mM KCl, 1 mM EDTA, 0.5 mM phenylmethylsulfonyl fluoride, and 2 mM dithiothreitol) at 4°C. The homogenate was centrifuged at 7,000 rpm for 10 min at 4°C. The supernatant was collected, and its protein content was determined using a dye-binding assay. Equal amounts of supernatant protein (40 µg/lane) were separated by SDS-PAGE, and then transferred onto polyvinylidene difluoride filters. The filters were probed using PLDδ-specific antibodies [39], followed by incubation with a second antibody conjugated to alkaline phosphatase. Activity of PLDδ was visualized by staining the blot for phosphatase activity [5].

### Measurements of Chlorophyll Content, Photosynthetic Activity, and Cell Death

After incubation in water or ABA, leaves were washed briefly in deionized water before analysis. Chlorophyll was extracted by incubating individual leaves in 80% acetone overnight. Chlorophyll content was determined spectrophotometrically at 663 and 646 nm in accordance with a previously described procedure [40]. Five replicates of each treatment for each genotype were analysed. Paired values were subjected to Student’s *t*-test to determine statistical significance. Chlorophyll fluorescence was analyzed using an imaging chlorophyll fluorometer, MAXI-Imaging Pulse-Amplitude (PAM) (Walz, Germany) [41]. The maximal quantum yield of photosystem II (PS II) photochemistry was measured in dark-adapted (20 minutes) samples on the basis of the initial fluorescence level (F_0_) and the maximal fluorescence level (F_m_), and expressed as F_v_/F_m_ = (F_m_-F_0_)/F_m_. The analysis was repeated twice.

Cell death, as indicated by a loss of plasma membrane integrity, was quantified spectrophotometrically by Evans blue staining of detached leaves, using a previously described method with minor modifications [42]. Briefly, detached leaves were incubated with 0.1% (w/v) Evans blue for 2 h with shaking, and then washed extensively to remove unbound dye. The leaves were ground into powder in liquid nitrogen. The tissue powder was incubated with 50% (v/v) methanol and 1% (w/v) SDS at 60°C for 30 min, and then centrifuged. For a control measurement of 100% cell death, the leaves were heated at 100°C for 5 min. Absorbance was measured at 600 nm. Five replicates of each treatment for each genotype were analysed. Paired values were subjected to Student’s *t*-test to determine statistical significance.

### Lipid Extraction and Analysis

Lipid extraction, ESI-MS/MS analysis, and lipid quantification were performed as described previously, but with minor modifications [35], [36]. Each sample contained two or three detached leaves with a pooled dry weight of 2–9 mg, and five replicates were analyzed for each genotype. To inhibit lipolytic activities, leaves were transferred immediately into 3 mL of isopropanol with 0.01% butylated hydroxytoluene at 75°C, and extracted several times with chloroform/methanol (2∶1) with 0.01% butylated hydroxytoluene, until all of the remaining leaves appeared white. Automated ESI-MS/MS analysis was performed by the Kansas Lipidomics Research Center (http://www.k-state.edu/lipid/lipidomics/) (Dataset S1). Data processing was performed as described previously [35]. The lipids in each class were quantified by comparison with two internal standards of that class. The Q-test was performed on the amount of lipid, and data from discordant samples were removed. Paired values were subjected to Student’s *t*-test to determine statistical significance.

Analysis of lipids using thin-layer chromatography (TLC) was performed as described previously, with minor modifications [43]. Total lipids were dissolved in chloroform, ensuring a constant relationship between the volume of chloroform and the dry weight of the plant materials. The equivalent amounts of lipid solution was separated into lipid classes by TLC [40∶5:2 (v/v/v) chloroform/methanol/formic acid]. Lipid spots were visualized by spraying with 0.05% (w/v) primuline reagent, and the positions of PA, PC, and PE were verified by comparison with standards (Avanti, http://www.avantilipids.com/). The analysis was repeated twice.

## Results

### The Effects of *n*-Butanol and NAE 12∶0 on ABA-promoted Leaf Senescence

**Figure 1:**
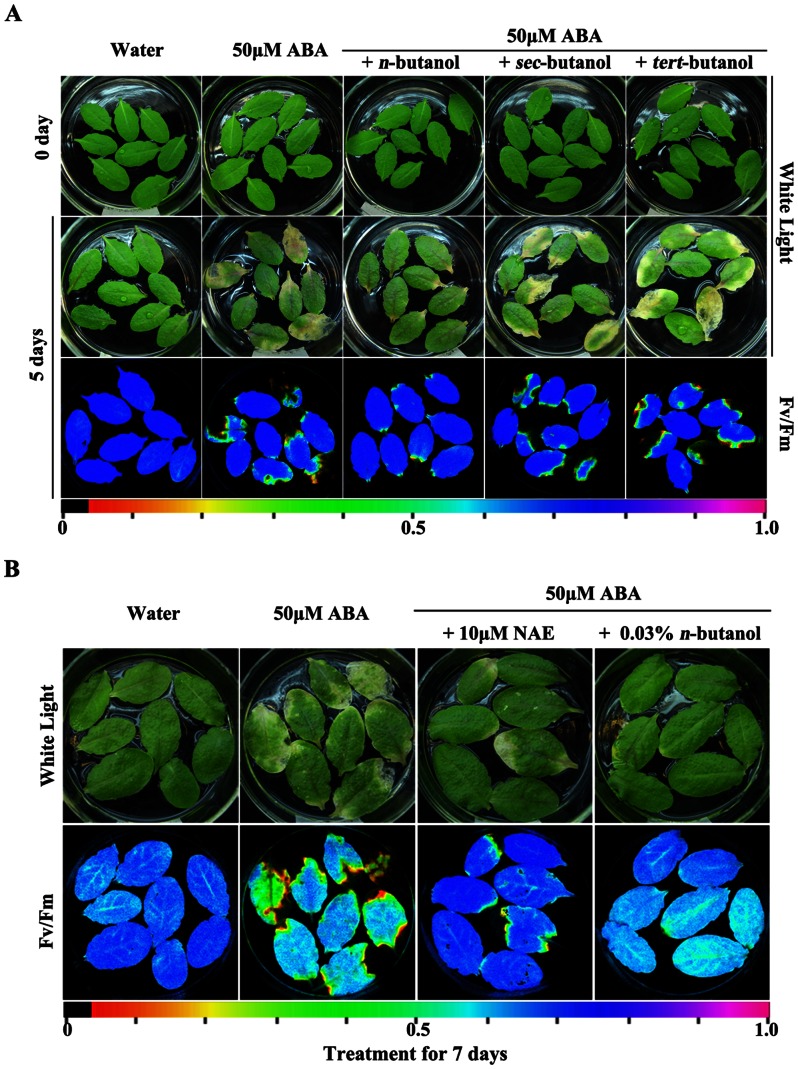
The effects of butanol and NAE on ABA-promoted leaf senescence. **A.** Leaves of *Arabidopsis* Col were detached and floated immediately on water, 50 µM ABA, or 50 µM ABA plus 0.03% *n*-, *sec-*, or *tert-*butanol. **B.** Leaves of *Arabidopsis* Col were detached and floated immediately on water, 50 µM ABA, 50 µM ABA plus 10 µM NAE, or 50 µM ABA plus 0.03% *n*-butanol. The color bar at the bottom indicates Fv/Fm values.

To investigate the potential roles of PLD-mediated PA in leaf senescence, we first examined the effects of *n*-, *sec-*, and *tert-*butanol on ABA-promoted leaf senescence. *n*-butanol specifically inhibits the cellular response induced by PLD-mediated PA, but *sec-* and *tert-*butanol have no effect [18], [21]. We floated detached leaves of *Arabidopsis* Col on either water, ABA, or ABA plus *n*-butanol, *sec-*butanol, or *tert-*butanol solution. We then assessed leaf senescence by observing leaf yellowing and measuring photosynthesis activity [44]. Photosynthesis activity was indicated by changes in F_v_/F_m_, the photochemical quantum efficiency of PS II. Detached leaves that were floated on water after 5 d did not show any visible yellowing (Figure 1A, middle panel) or changes in photosynthesis (Figure 1A, bottom panel). Leaves that were treated with ABA showed more yellowing and substantially reduced photosynthesis activity as compared with the control, which confirmed that ABA promoted leaf senescence. Less yellowing and higher photosynthesis activity was seen in the leaves that were treated with ABA plus *n*-butanol as compared with those treated with ABA alone. Leaves that were treated with ABA plus *sec-*butanol or *tert-*butanol showed similar effects to those treated with ABA alone. Leaves that were treated with only *n*-butanol showed no effects to senescence (Figure S1). Leaves of *Arabidopsis* WS that treated with ABA and ABA plus *n-*butanol showed similar effects to those of *Arabidopsis* Col (Figure S2). The results indicated that application of *n*-butanol attenuated ABA-promoted senescence but *sec-* and *tert-*butanol had no effect. These findings suggested that PLD-mediated PA has a role in the retardation of ABA-promoted senescence.

To investigate the potential roles of PLD isoforms other than PLDα1 in leaf senescence, we then compared the effects of *n*-butanol and NAE 12∶0 on ABA-promoted leaf senescence. *n*-butanol suppresses the formation of PA by any isoform of PLD [18], [45], whereas NAE only inhibits PLDα1 activity [22], [24]. Treatment with ABA plus NAE resulted in more yellowing and lower photosynthesis activity than in leaves treated with water alone, but the effects were less than those seen in leaves treated with ABA alone (Figure 1B). Leaves that treated with only NAE showed no effects to senescence (Figure S1). Leaves of *Arabidopsis* WS that treated with ABA and ABA plus NAE showed similar effects to those of *Arabidopsis* Col (Figure S2). The results indicated that application of NAE attenuated ABA-promoted senescence to some extent but not completely. Furthermore, treatment with ABA plus NAE showed more yellowing and lower photosynthesis activity than in leaves treated with ABA plus *n*-butanol. (Figure 1B). These findings suggested that PLDα1-mediated PA plays only a partial role in the retardation of ABA-promoted senescence and that PA generated by other PLDs might also be involved in the process. Given that PLDδ is one of the two most abundant PLDs, we chose it as our candidate PLD for further study.

### The Expression of PLDδ during Hormone-promoted Leaf Senescence

**Figure 2:**
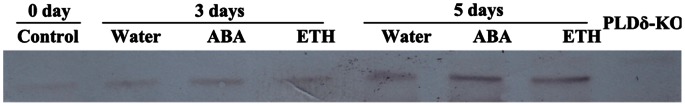
Changes in PLDδ abundance in detached *Arabidopsis* leaves floated on water, ABA, or ethylene. Total protein (40 µg) was extracted from WS leaves treated with either water, 50 µM ABA, or 50 µM ethephon (ETH) for 0, 3, and 5 days and separated by SDS-PAGE. PLDδ was detected by western blotting using a PLDδ-specific antibody. Protein from PLDδ-KO leaves that underwent no additional treatment is shown as a negative control.

We examined levels of PLDδ protein in detached *Arabidopsis* WS leaves that had or had not been treated with the hormone ABA or ethylene for either 3 or 5 days. Assays of comparable leaves immediately after harvesting from *Arabidopsis* WS plants provided a control. We used immunoblotting with a PLDδ-specific antibody to determine levels of PLDδ [39]. Levels of PLDδ were higher in detached leaves that were floated on either water or water that contained ABA or ethylene than in control leaves. Moreover, levels of PLDδ were higher in ABA- or ethylene-treated detached leaves than in those detached at same time but exposed only to water (Figure 2). Together, the results described above indicated that exposure to hormone induced the expression of PLDδ more than detachment alone, and implied that PLDδ might have a role in hormone-induced senescence. We focused on detachment- and ABA-induced senescence in subsequent studies.

### The Effects of PLDδ Suppression on Detachment-induced and ABA-promoted Senescence

**Figure 3:**
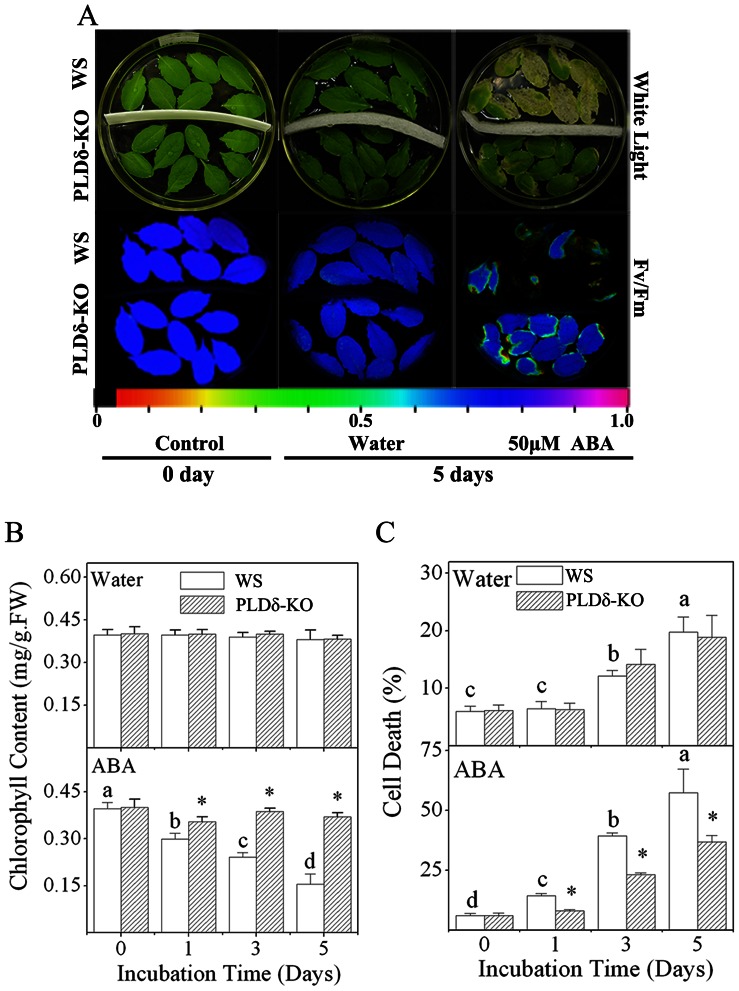
Senescence of detached leaves from WS and PLDδ-KO plants. Leaves detached from WS and PLDδ-KO plants were treated with sterile water or 50 µM ABA for 5 days. Retardation of ABA-promoted senescence was compared between PLDδ-KO and WS leaves. **A.** Yellow coloration (top panel) or low Fv/Fm values for variable fluorescence (bottom panel) indicated the degree of senescence. The color bar at the bottom indicates Fv/Fm values. **B.** Effects of exogenous ABA on the chlorophyll content of leaves from WS and PLDδ-KO plants. FW, fresh weight. Values are means ± SDs (*n* = 5). **C.** Effects of exogenous ABA on cell death in leaves from WS and PLDδ-KO plants. Cell death was determined spectrophotometrically using Evan’s blue staining. Values are means ± SDs (*n = *5). Values with different letters are significantly different (*p*<0.05). “*” indicates that the value is significantly different from that for WS under the same condition (*p*<0.05).

Leaf senescence did not show senescence until floated in water for 15 days and there was not difference between WS and PLDδ-KO plants (Figure S1, a and b). To investigate the potential role of PLDδ in leaf senescence, we floated detached leaves of WS and PLDδ-KO plants on either water or an ABA solution for 5 days and conducted a detailed assessment of their senescence on the basis of not only the changes in the properties mentioned above, but also the changes in chlorophyll content and cell death. Detached leaves that were floated on water did not show any visible yellowing, or changes in photosynthesis (Figure 3A) or chlorophyll content (Figure 3B) as compared with control leaves. Nonetheless, they did show a significant increase in cell death (Figure 3C), which indicated a slow senescence. The finding that there was no difference between WS and PLDδ-KO in water-floated leaves in terms of yellowing, changes in photosynthesis, chlorophyll content, or cell death indicated that PLDδ is not involved in detachment-induced senescence.

In comparison with the leaves floated on water, leaves that were treated with ABA showed not only more yellowing and substantially reduced photosynthesis activity, but also dramatically reduced chlorophyll contents and increased rates of cell death (Figure 3B and 3C). However, the leaves of PLDδ-KO plants showed significantly less yellowing, higher photosynthesis activity, higher chlorophyll contents, and less cell death than those of WS plants following treatment with ABA (Figure 3B and 3C). Complementation of PLDδ in PLDδ-RC plants restored the ABA-promoted senescence to the same levels as that in WS plants (Figure S3). These results indicated that ABA-promoted leaf senescence was retarded in PLDδ-KO plants, which suggests that PLDδ plays a positive role in ABA-promoted leaf senescence.

### Profiles of Molecular Species of Membrane Lipids in WS and PLDδ-KO Plants during Detachment-induced and ABA-promoted Senescence

**Figure 4:**
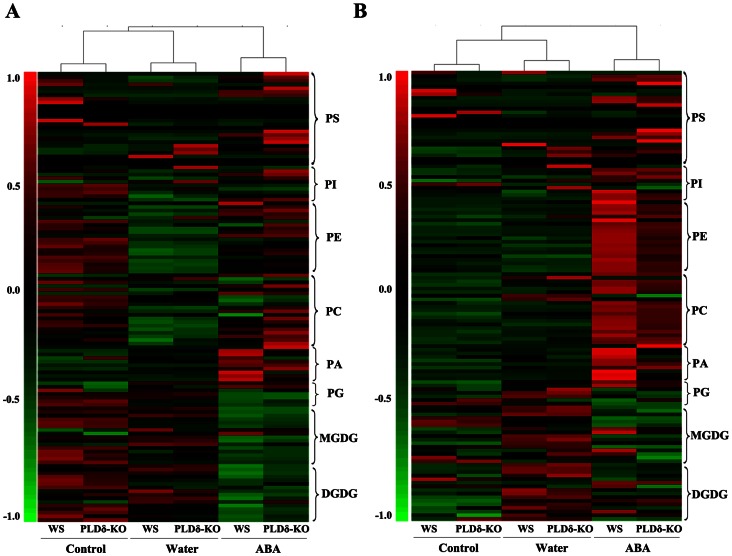
Hierarchal clustering analysis of lipid molecular species during detachment-induced and ABA-promoted senescence. Leaves that had been detached from WS and PLDδ-KO plants were treated with sterile water or 50 µM ABA for 5 days. **A.** Absolute levels (nmol/mg dry weight) of lipid molecular species. **B.** Relative levels (mol%) of lipid molecular species. Each colored bar within a column represents the lipid molecular species in the indicated plants and treatments. The mean value of the same lipid molecular species in different plants and treatments in a row was calculated. The row-wise mean is subtracted from the values in each row of data, so that mean value of each row is zero. Each colored bar within a row represents the relative change from the mean center of each lipid species. Lipid species in the indicated lipid classes were organized using class (as indicated), total acyl carbons (in ascending order within a class), and total double bonds (in ascending order with class and total acyl carbons).

To understand better how PLDδ, in particular PLDδ-mediated PA, functions in senescence, we profiled the changes in membrane lipids that occurred in detached leaves incubated in water or ABA solution for 5 days. We used a lipidomics approach based on ESI-MS/MS to profile the lipids [35], [36]. We identified quantitatively more than 125 molecular species of polar glycerolipids, which included six head-group classes of phospholipids–PC, phosphatidylethanolamine (PE), phosphatidylinositol (PI), phosphatidylserine (PS), PA, phosphatidylglycerol (PG)–and two head-group classes of galactolipids, MGDG and DGDG (Dataset S1). Each molecular species was identified in relation to the total number of acyl carbon atoms and double bonds [35]. Changes in both the absolute levels of these lipids (nmol/mg dry weight), which reflect lipid degradation, and the relative levels of these lipids (mol%), which can reflect the interconversion among lipids, were visualized using hierarchal clustering analysis (Figure 4A and 4B). The total amounts of lipid and the average levels of molecular species in each head group class are shown in Tables 1 and 2, respectively.

**Table 1:** Total lipids in leaves of WS and PLDδ-KO plants during detachment-induced and ABA-promoted senescence.

Treatment	Genotype	Lipid/dry weight (nmol/mg)		RC
		0 day	5 days	(%)
Water	WS	302.2±28.3^a^	183.6±16.0^b^	−39.2
	PLDδ-KO	283.5±38.9^a^	179.3±15.7^b^	−36.8
ABA	WS	302.2±28.3^a^	102.5±15.4^b^	−66.1
	PLDδ-KO	283.5±38.3^a^	137.4±19.1^b*^	−51.5

The relative change (RC) in the levels of lipids from 0 to 5 days is the percentage value for the significant difference between the values at day 0 and 5 days over the value at day 0. Values in the same row with different letters are significantly different (*P*<0.05). “*” indicates that the value is significantly different from that of the WS under the same condition (*p*<0.05). Values are means ± SDs (*n* = 5).

**Table 2:** Amount of lipid in each head group class in leaves of WS and PLDδ-KO plants during detachment-induced and ABA-promoted senescence.

Lipid	Treatment	Genotype	Lipid/dry weight (nmol/mg)		RC(%)
class			0 day	5 days	
**PG**	Water	WS	12.9±1.9^a^	7.2±0.9^b^	−44.2
		PLDδ-KO	12.7±5.0^a^	7.4±0.5^b^	−41.7
	ABA	WS	12.9±1.9^a^	3.7±1.2^b^	−71.3
		PLDδ-KO	12.7±5.0^a^	4.8±0.6^b^	−62.2
**PC**	Water	WS	16.7±1.6^a^	11.4±1.3^b^	−31.7
		PLDδ-KO	15.2±4.7^a^	11.8±2.1^a^	–
	ABA	WS	16.7±1.6^a^	13.4±1.7^b^	−19.8
		PLDδ-KO	15.2±4.7^a^	16.5±2.5^a^	−
**PE**	Water	WS	9.9±1.1^a^	6.6±0.6^b^	−33.3
		PLDδ-KO	9.2±2.8^a^	6.7±1.2^a^	–
	ABA	WS	9.9±1.1^a^	10.4±1.8^a^	–
		PLDδ-KO	9.2±2.8^a^	11.4±0.1^a^	–
**PI**	Water	WS	2.4±0.6^a^	1.8±0.5^a^	–
		PLDδ-KO	2.6±0.5^a^	2.4±0.6^a^	–
	ABA	WS	2.4±0.6^a^	3.0±0.6^a^	–
		PLDδ-KO	2.6±0.5^b^	3.6±0.5^a^	38.5
**PA**	Water	WS	0.07±0.0^a^	0.11±0.1^a^	–
		PLDδ-KO	0.09±0.0^a^	0.09±0.1^a^	–
	ABA	WS	0.07±0.0^b^	0.53±0.0^a^	657
		PLDδ-KO	0.09±0.0^b^	0.15±0.0^a^*	66.7
**PS**	Water	WS	0.35±0.1^a^	0.41±0.1^a^	–
		PLDδ-KO	0.20±0.1^b^	0.91±0.6^a^	355
	ABA	WS	0.35±0.1^b^	0.61±0.2^a^	74.3
		PLDδ-KO	0.20±0.1^b^	0.80±0.1^a^	300
**MGDG**	Water	WS	235.6±17.7^a^	128.3±13.2^b^	−45.5
		PLDδ-KO	217.8±25.9^a^	122.8±10.8^b^	−43.6
					
	ABA	WS	235.6±17.7^a^	57.64±10.0^b^	−75.5
		PLDδ-KO	217.8±25.9^a^	84.5±13.9^b^*	−61.2
**DGDG**	Water	WS	31.7±4.8^a^	27.8±2.8^a^	–
		PLDδ-KO	31.9±6.9^a^	27.4±1.3^a^	–
	ABA	WS	31.7±4.8^a^	13.1±3.0^b^	−58.7
		PLDδ-KO	31.9±6.9^a^	19.4±2.7^b^*	−39.2

The relative change (RC) in lipids from 0 to 5 days is the percentage value for the significant difference between the values at day 0 and 5 days over the value at day 0. Values in the same row with different letters are significantly different (*P*<0.05). “*” indicates that the value is significantly different from that of the WS under the same condition (*p*<0.05). Values are means ± SD (*n* = 5).

An overview of the data shown in Figure 4 revealed complex and considerable changes in lipid molecular species during leaf senescence. Changes in the lipid profile during ABA-promoted senescence were much more dramatic than those during detachment-induced senescence, in terms of both the levels (absolute value, Figure 4A) and composition (relative value, Figure 4B) of lipids. The levels of most lipids declined, although some increased in terms of both their absolute and relative levels. This suggested that the degradation of distinct lipids was not synchronous, and that interconversions among lipids might occur. The differences between control leaves and leaves that had undergone ABA-promoted senescence were greater than those between control leaves and leaves that had undergone detachment-induced senescence. The differences between WS and PLDδ-KO leaves following ABA treatment were greater than those between WS and PLDδ-KO leaves floated on water (Figure 4A and 4B). These results suggested that ABA treatment affected lipid degradation, and that elimination of PLDδ affected lipid degradation during ABA-promoted senescence in particular. Detailed data mining of the changes in membrane lipids and the functional characterization of PLDδ during leaf senescence are described below.

### Different Effects of Detachment-induced Senescence on Plastidic and Extraplastidic Lipids

Levels of leaf membrane lipid decreased significantly during the detachment-induced senescence of WS leaves (Table 1). Total lipids decreased by 39.2%, from 302.2 nmol/mg in nonsenescent leaves to 183.6 nmol/mg within 5 days after the commencement of detachment-induced senescence. Levels of PG, PC, PE, PI, and MGDG all decreased significantly. As shown in Table 2, levels of MGDG, the most abundant class of plastidic lipid, decreased by 45.5% (from 235.6 nmol/mg in nonsenescent leaves to 128.3 nmol/mg after detachment-induced senescence). The decreases in both the amount and percentage of MGDG were the highest among all the lipid classes. Levels of PA and PS, two extraplastidic lipids, remained unchanged. These data implied that changes in membrane lipids differed between plastidic and extraplastidic lipids.

To compare lipid degradation between plastidic and extraplastidic membranes further, we analyzed changes in the levels of molecular species of PG. In *Arabidopsis*, PG includes four molecular species, namely 34∶1 (total carbon number:double bond number) PG, 34∶2 PG, 34∶3 PG, and 34∶4 PG [35]. Whereas 34∶4 PG, which harbors a 16∶1 acyl chain, is part of the plastidic membrane, both 34∶1 PG and 34∶2 PG are extraplastidic lipids. Of the two molecules that correspond to 34∶3 PG, one contains a 16∶1 acyl chain and is part of the plastidic membrane, whereas the other is extraplastidic [46]. During detachment-induced senescence in WS leaves, the decrease in the percentage of 34∶4 PG was more than that of 34∶3 PG, and the decrease in 34∶3 PG was more than that of either 34∶2 PG or 34∶1 PG combined (Table 3). In other words, the degradation of PG molecular species in plastidic membranes was significantly greater than that in extraplastidic membranes. These results indicated that most of the dramatic degradation of lipids during detachment-induced senescence occurred in plastids. During detachment-induced senescence, the patterns of changes in membrane lipids were similar in PLDδ-KO leaves and WS leaves (Tables 1, 2, and 3). There were no differences in lipid levels between WS and PLDδ-KO leaves. Combined with the physiological observations mentioned above, these results demonstrated that PLDδ was not involved in the regulation of detachment-induced senescence.

**Table 3:** Levels of PG molecular species in leaves of WS and PLDδ-KO plants during detachment-induced and ABA-promoted senescence.

PG	Treatment	Genotype	Lipid/dry weight (nmol/mg)		RC
species			0 day	5 days	(%)
34∶1	Water	WS	0.65±0.19^a^	0.69±0.09^a^	–
		PLDδ-KO	0.69±0.33^a^	0.73±0.25^a^	–
	ABA	WS	0.65±0.19^a^	0.20±0.23^b^	−69.2
		PLDδ- KO	0.69±0.33^a^	0.19±0.09^b^	−72.5
34∶2	Water	WS	0.98±0.32^a^	0.93±0.21^a^	–
		PLDδ- KO	0.93±0.15^a^	0.88±0.19^a^	–
	ABA	WS	0.98±0.32^a^	0.44±0.26^b^	−55.1
		PLDδ- KO	0.93±0.15^a^	0.41±0.18^b^	−55.9
34∶3	Water	WS	2.80±0.17^a^	1.65±0.41^b^	−41.1
		PLDδ- KO	3.07±0.47^a^	1.50±0.17^b^	−51.1
	ABA	WS	2.80±0.17^a^	0.86±0.30^b^	−69.3
		PLDδ- KO	3.07±0.47^a^	1.30±0.32^b^	−57.7
34∶4	Water	WS	8.18±1.56^a^	3.50±0.42^b^	−57.2
		PLDδ- KO	7.84±1.67^a^	3.84±0.33^b^	−51.0
	ABA	WS	8.18±1.56^a^	1.67±0.42^b^	−79.6
		PLDδ- KO	7.84±1.67^a^	2.34±0.30^b^*	−70.2

The relative change (RC) in lipids from 0 to 5 days is the percentage value for the significant difference between the values at day 0 and 5 days over the value at day 0. Values in the same row with different letters are significantly different (*P*<0.05). “*” indicates that the value is significantly different from that of the WS under the same condition (*p*<0.05). Values are means ± SD (*n* = 5).

### Changes in Plastidic Lipids during ABA-promoted Senescence

During ABA-promoted senescence, the amount of total membrane lipids in WS leaves declined by 66.1% (from 302.2 nmol/mg to 102.5 nmol/mg), which was much more than the 39.2% decrease recorded for leaves exposed to water alone (Table 1). Most of the decrease in lipid levels could be attributed to reduced levels of plastidic lipids (Table 2). For example, levels of MGDG decreased by 75.5% (from 235.6 nmol/mg to 57.6 nmol/mg), and levels of DGDG decreased by 58.7% (from 31.7 nmol/mg to 13.1 nmol/mg). In contrast with the levels of plastidic lipids, levels of PC decreased by only 19.8% (from 16.7 nmol/mg to 13.4 nmol/mg), levels of PE maintained unchanged, and levels of PI, PA, and PS all increased. Total PG decreased by 71.3% (from 12.9 nmol/mg to 3.7 nmol/mg). With respect to the molecular species of PG (Table 3), 34∶4 PG, 34∶1 PG, 34∶2 PG, and 34∶3 PG deceased by 79.6%, 69.3%, 55.1%, and 69.2%, respectively. The decrease in levels of 34∶4 PG was significantly more than those for 34∶1 PG, 34∶2 PG, and 34∶3 PG. These results indicated that the degradation of lipids, in particular plastidic lipids, occurred during ABA-promoted senescence, and that differences in the relative abundances of plastidic and extraplastidic lipids were amplified during ABA-promoted senescence.

### Attenuation of the Decrease in Levels of Plastidic Lipids in PLDδ-KO Leaves during ABA-promoted Senescence

During ABA-promoted senescence in PLDδ-KO leaves, the amount of total lipids declined by 51.5% (from 283.5 nmol/mg to 137.4 nmol/mg), which was significantly less than the decrease of 66.1% observed in WS (Table 1). Levels of MGDG, DGDG, and PG were significantly lower in ABA-treated leaves than in those exposed to water, whereas the levels of PC, PE, PI, PA, and PS were higher than or similar to those in leaves exposed to water (Table 2). These data indicated that decreases in lipid levels also occurred in PLDδ-KO plants, but these decreases occurred only among plastidic lipids during ABA-promoted senescence. In contrast, the levels of MGDG and DGDG were significantly higher in PLDδ-KO leaves than in WS leaves (Table 2). The levels of the plastidic lipids 34∶4 PG and 34∶3 PG were higher in leaves of PLDδ-KO plants than in leaves of WS. Levels of the extraplastidic lipids 34∶2 PG and 34∶1 PG in PLDδ-KO plants were closer to those in WS. These results indicated that the degradation of plastidic lipids was attenuated in PLDδ-KO leaves during ABA-promoted senescence. This suggested that retardation of ABA-promoted senescence in PLDδ-KO plants might result from attenuation of plastidic lipid degradation, and that delay of membrane degradation might retard senescence.

### Changes in Levels of Extraplastidic Lipids during ABA-promoted Senescence

The pattern of changes in levels of extraplastidic lipids in WS leaves during ABA-promoted senescence was more complicated than that during detachment-induced senescence. During ABA-promoted senescence, levels of PC decreased significantly (albeit less than during detachment-induced senescence), levels of PE and PI remained unchanged, levels of PS increased slightly by an amount comparable to that observed during detachment-induced senescence, and levels of PA increased much more than during detachment-induced senescence (Table 2). In other words, whereas the observed trend of decreased levels of PE, PC, and PI during detachment-induced senescence became less evident or disappeared altogether during ABA-promoted senescence, the increase in PA during detachment-induced senescence was even more evident during ABA-promoted senescence of WS leaves. Considering the accelerated decrease in plastidic lipids during ABA-promoted senescence, these results indicated that ABA- and detachment-induced senescence have opposite effects on plastidic and extraplastidic lipids, with degradation of extraplastidic membranes occurring later than degradation of plastidic membranes during ABA-promoted senescence.

### Changes in PA Levels during ABA-promoted Senescence

During ABA-promoted senescence, PA and PS were only two lipid classes for which levels increased significantly in leaves from both WS and PLDδ-KO plants. However, the observation that PS levels in WS resembled those in PLDδ-KO leaves (Table 2), suggested that elimination of PLDδ did not affect PS levels, which indicated that PS was not involved in the PLDδ-mediated retardation of senescence. The effect of PLD might relate to its structural role or the effects of its product, PA [47]. To investigate how PLDδ functions in ABA-promoted senescence, we analyzed changes in the levels of PA under two conditions of senescence in both plant genotypes. Whereas levels of PA increased only slightly during detachment-induced senescence, a 6-fold increase was evident following ABA-promoted senescence. Moreover, retardation of ABA-promoted senescence reduced PA levels (Table 2). These results indicated that changes in PA levels were associated tightly with ABA-promoted senescence. Whereas levels of PA increased by 0.5 nmol/mg in WS plants (from 0.1 to 0.6 nmol/mg), they increased by only 0.1 nmol/mg (from 0.1 to 0.2 nmol/mg) in PLDδ-KO plants. This suggested that at concentrations of approximately 0.4 nmol/mg, the formation of PA is suppressed by approximately 85% in plants in which PLDδ has been ablated. These results indicated that the additional PA in WS plants was derived mainly from PLDδ-mediated hydrolysis. Therefore, limited availability of PA might account for the retardation of ABA-promoted senescence in PLDδ-KO plants.

Both PLDα1 and PLDδ are key members of the PLD family in plants. Although the symptoms of delayed leaf senescence from plants with reduced levels of PLDα1 was reported [27],if and how their lipids and PA in particular changed was not examined. The retardation of ABA-promoted senescence under both *n*-butanol and NAE treatments (Figure 1) implied that PLDα1-mediated PA was involved in the process. We used TLC to measure PA levels in leaves from Col and PLDα1-antisense (PLDα1-AS) *Arabidopsis* plants and found that levels of PA were lower in leaves of PLDα1-AS plants than in leaves from Col plants after ABA treatment (Figure S4). These lines of evidence indicated that PLDα1-mediated PA plays important roles in ABA-promoted senescence and suggested that both PLDα1 and PLDδ might function in ABA-promoted senescence through the same mechanism, which involves the regulation of PA formation. The results also demonstrated that limiting the formation of PA retards ABA-promoted leaf senescence.

### Changes in PC Levels during ABA-promoted Senescence

Given that PLD may use different phospholipids as substrates [14], we examined changes in phospholipids to identify potential substrates of PLDδ during ABA-promoted senescence. After treatment with ABA for 5 days, the levels of PE and PI did not decrease in WS leaves and were comparable between leaves of WS and PLDδ-KO plants (Table 2). Given our demonstration that PS was not involved in ABA-promoted senescence, we conclude that PE, PI, and PS cannot be the substrates of PLDδ. However, the significant decrease in levels of PC in WS leaves and the observation that this decrease was suppressed in leaves from PLDδ-KO plants together suggested that PC is hydrolyzed by PLDδ to generate PA.

## Discussion

The metabolism of organelle membranes plays a crucial role during leaf senescence. The general involvement of PLD and its product PA in the deterioration of senescing leaves has been reported widely [2], [13], with PLDα1 having been the first PLD identified to function in ABA-promoted senescence [27]. Here we report that the complementary use of biochemical and genetic approaches demonstrates a positive role for PLDδ in ABA-promoted leaf senescence through the regulation of lipid degradation. Our data suggest that PLDδ uses PC as its preferred substrate to produce PA. Not only was PLDδ responsible for most of the increase in levels of PA, but PLDα1 also contributed to the formation of PA during ABA-promoted senescence. We also performed detailed analysis of membrane lipid degradation during leaf senescence. Plastidic and extraplastidic lipids showed different patterns of degradation. The levels of all plastidic lipids decreased, with the most extensive degradation occurring in levels of MGDG. Changes in levels of plastidic lipids were correlated closely with the degree of leaf senescence, with the degradation of plastidic lipids accelerated during ABA-promoted senescence, and both delayed and attenuated degradation observed during ABA-promoted senescence in PLDδ-KO leaves.

It is known that PLDδ functions in various important environmental responses [15], including responses to dehydration, stomatal closure, salinity, and freezing, and in programmed cell death in response to H_2_O_2_ [16], [37], [48]–[50]. We report here the first evidence that PLDδ is also involved in hormonally regulated senescence. Three mechanisms have been proposed for how PLDδ functions in cellular processes. The first of these suggests that PLDδ might act as a protein bridge that links the plasma membrane and microtubules, and thus can convey hormonal and/or environmental signals from outside the cell to inside the cell [51]. The second suggests that PLDδ functions in signal transduction through its product, PA, which functions as a second messenger [37]. The third possible mechanism proposes that PLDδ affects membrane composition by hydrolyzing phospholipids to PA [48], [49], which serves as a structural component and favors the formation of the nonlamellar phase in membranes [35], [52], [53]. By investigating the effects of both the inhibitors *n*-butanol and NAE 12∶0 and the PLDδ-KO mutant, we obtained data that imply a lipolytic role for PLDδ and demonstrate that the involvement of PLDδ in ABA-promoted senescence is mediated, at least in part, by the third mechanism. The stoichiometry of PA formation indicates that PLDδ-mediated hydrolysis contributes approximately 20% of the total PA formed under conditions of freezing [49] and 10–25% of the total PA formed under conditions of salinity [16]. In contrast, the contribution of PLDδ-derived PA is as high as 85% during ABA-promoted senescence. This suggests strongly that suppression of PLDδ blocks the degradation of membrane lipids and consequently retards ABA-promoted senescence. The low PA levels resulting from suppression of PLDδ could have propensity for membrane integrity and thus might contribute to senescence attenuation.

In plants, the PLD family comprises 12 members, whereas only two PLDs have been identified in animals [14]. In contrast, animals show greater diversity than plants in relation to other phospholipase families, such as phospholipase A and phospholipase C. It has been hypothesized that the basis for the differences between plant and animal PLDs is that PLD might play a more diverse and important role in plants than in other organisms [54]. This makes it especially interesting to elucidate the unique and redundant functions of plant PLDs. For example, the functions of the PLDζ1 and PLDζ2 proteins, which share 74% sequence similarity [14], apparently overlap during phosphate-limited growth in *Arabidopsis*. Only a double mutant that is deficient in both of the proteins shows defective root elongation; neither of the single mutants that lack PLDζ1 or PLDζ2 have this phenotype. The PLDα1 and PLDδ isoforms are the most abundant PLDs in plants. Even though they are not related closely in structural terms and differ in their requirements for activity and subcellular localization [14], both PLDα1 and PLDδ function in the same manner during ABA-promoted leaf senescence. Suppression of either of the genes that encode these proteins causes the retardation of senescence with associated effects on membrane metabolism. Notwithstanding these similarities, our data suggest that the functions of PLDα1 and PLDδ do not overlap. Their contributions to ABA-promoted senescence are unique, because PLDδ does not compensate for PLDα1 function in PLDα1-deficient plants, and vice versa.

It is accepted widely that the process of leaf senescence is regulated tightly. At the biochemical level, the degradation of chlorophyll is one of the key initial events [11], although PLD-mediated metabolism is also thought to occur early during the process [13]. With respect to organelle membranes, degradation occurs first at the thylakoid membrane and last at the plasma membrane [34]. Our experiments revealed that senescence had different effects on the metabolism of plastidic and extraplastidic lipids: whereas senescence primarily caused degradation of plastidic lipids, levels of extraplastidic lipids decreased only slightly, remained unchanged, or even increased substantially, as in the case of PA (Table 2). These differences were exemplified by the far greater decrease in PG molecular species among plastidic lipids than among extraplastidic lipids (Table 3). Given that breakdown of extraplastidic lipids is inevitable during the final stage of leaf senescence, our data suggest that the degradation of plastidic lipids precedes that of explastidic lipids.

Another interesting question that arises concerns why levels of PA increased so substantially during ABA-promoted senescence. A model to explain the positive roles of PLDα1 and PLDδ during ABA-promoted senescence, as well as the effects of PA on metabolism, is proposed here. It is known that PA is a major intermediate during lipid metabolism, with both lipid degradation and lipid synthesis being regulated by the size of the PA pool [55]. During ABA-promoted senescence, the accelerated degradation of membrane lipids causes more PA to accumulate. Most of the PA produced through metabolism is produced via PLD hydrolysis. Plastidic lipid MGDG and DGDG might get into PLD-mediated hydrolysis of explastidic lipids through this degradation pathway. They breakdown into diacylglycerols (DAG) by glycolipases [35], [56], transporte to ER and then turnover into PC [55]. Given that suppression of PLDα1 or PLDδ partly blocks the formation of PA, and thus reduces the rate of lipid degradation, reduced rates of lipid degradation might prolong membrane integrity and eventually retard senescence.

In summary, we have shown that PLDδ positively regulates the degradation of membrane lipids during ABA-promoted senescence and have described detailed changes in the levels of membrane lipids during leaf senescence. Suppression of PLDδ attenuates lipid metabolism through the formation of PA, and thus retards ABA-promoted senescence. The PLDα1 and PLDδ isoforms have similar, but unique, roles in leaf senescence. We propose that blocking membrane lipid metabolism by restricting the formation of PA might delay leaf senescence.

## Supporting Information

Figure S1 **The effects of water, butanol, and NAE on leaf senescence. a,** leaves of *Arabidopsis* WS were detached and floated immediately on water for indicated days. **b**, leaves of WS and PLDδ-KO *Arabidopsis* were detached and floated immediately on water for indicated days. **c**, leaves of *Arabidopsis* WS were detached and floated immediately on water, 10 µM NAE, or 0.03% *n*-butanol for indicated days. The color bar at the bottom indicates Fv/Fm values.
(TIF)

Figure S2 **The effects of butanol and NAE on ABA-promoted leaf senescence.** Leaves of *Arabidopsis* WS were detached and floated immediately on water, 50 µM ABA, 50 µM ABA plus 10 µM NAE, or 50 µM ABA plus 0.03% *n*-butanol for 7 days. The color bar at the bottom indicates Fv/Fm values.
(TIF)

Figure S3 **Senescence of detached leaves from WS, PLDδ-KO, and PLDδ-RC plants.** Leaves detached from WS, PLDδ-KO, and PLDδ-RC plants were treated with sterile water or 50 µM ABA for 3 days. ABA-promoted senescence was compared among WS, PLDδ-KO, and PLDδ-RC leaves. Yellow coloration (top panel) or low Fv/Fm values for variable fluorescence (bottom panel) indicated the degree of senescence. The color bar at the bottom indicates Fv/Fm values.
(TIF)

Figure S4 **Analysis of PA, PE, and PC in leaves of WT and PLDα1-AS plants during detachment-induced and ABA-promoted senescence, using TLC.** Lipid spots were visualized using primuline, and the identification of PA, PC, and PE was verified by comparison of their migration with standards (indicated using arrows).
(TIF)

Dataset S1 **Amount (nmol/mg dry weight ) of all lipid species detected.** Five replicates of each treatment for each phenotype were carried out and analyzed by mass spectrometry. Highlighted values were the discordant data (Q-test). “ave” = average, “stdev” = standard deviation.
(XLS)
