# Supplementary figures and images for: Lipid Profiling Demonstrates That Suppressing Arabidopsis Phospholipase Dδ Retards ABA-Promoted Leaf Senescence by Attenuating Lipid Degradation
Source: PLoS One. 2013 Jun 7;8(6):e65687. doi: 10.1371/journal.pone.0065687 (PMC3676348; doi:10.1371/journal.pone.0065687)

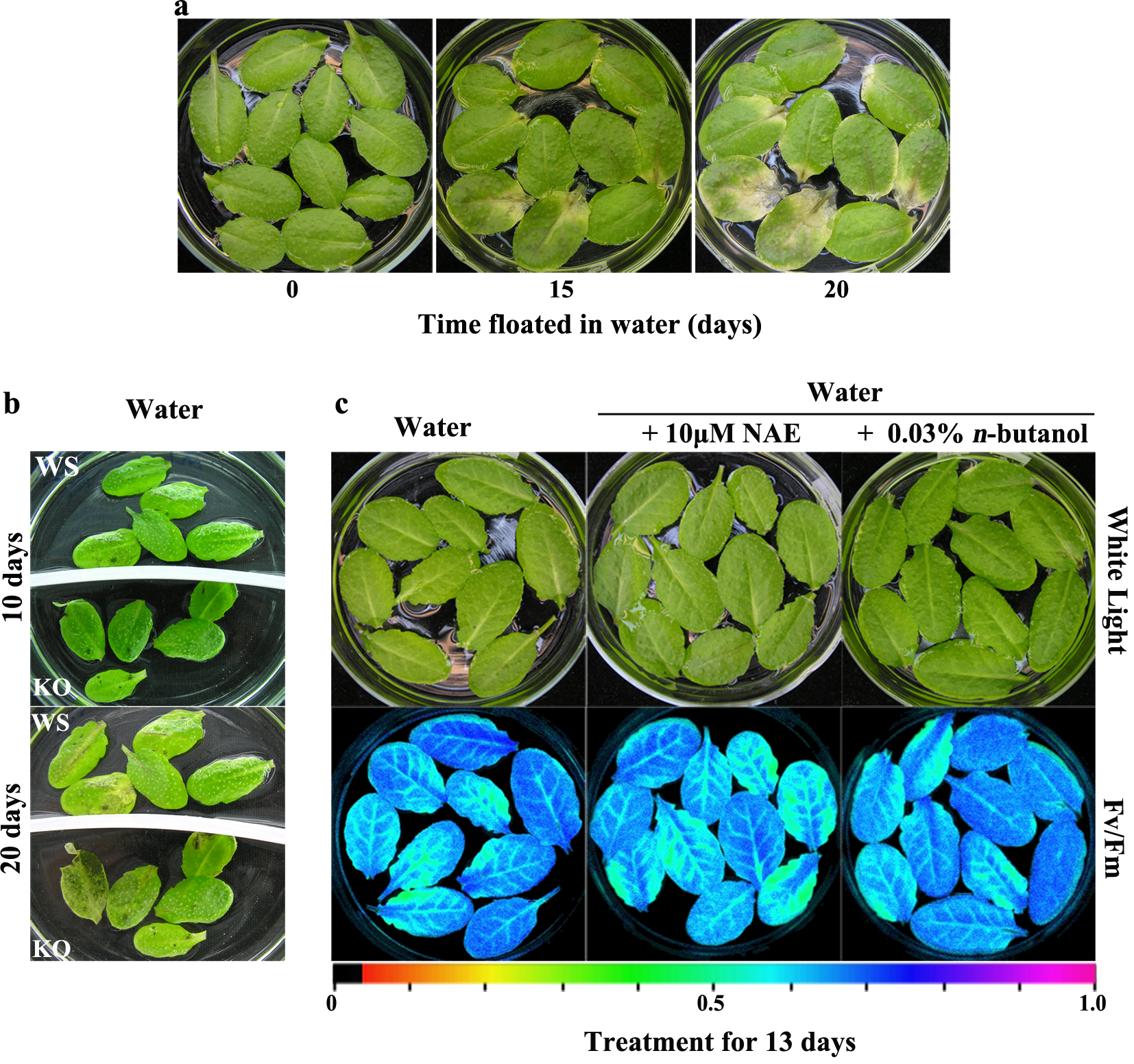

Supplement: Figure S1 — The effects of water, butanol, and NAE on leaf senescence. a, leaves of Arabidopsis WS were detached and floated immediately on water for indicated days. b, leaves of WS and PLDδ-KO Arabidopsis were detached and floated immediately on water for indicated days. c, leaves of Arabidopsis WS were detached and floated immediately on water, 10 µM NAE, or 0.03% n-butanol for indicated days. The color bar at the bottom indicates Fv/Fm values. (TIF) [file pone.0065687.s001.tif]

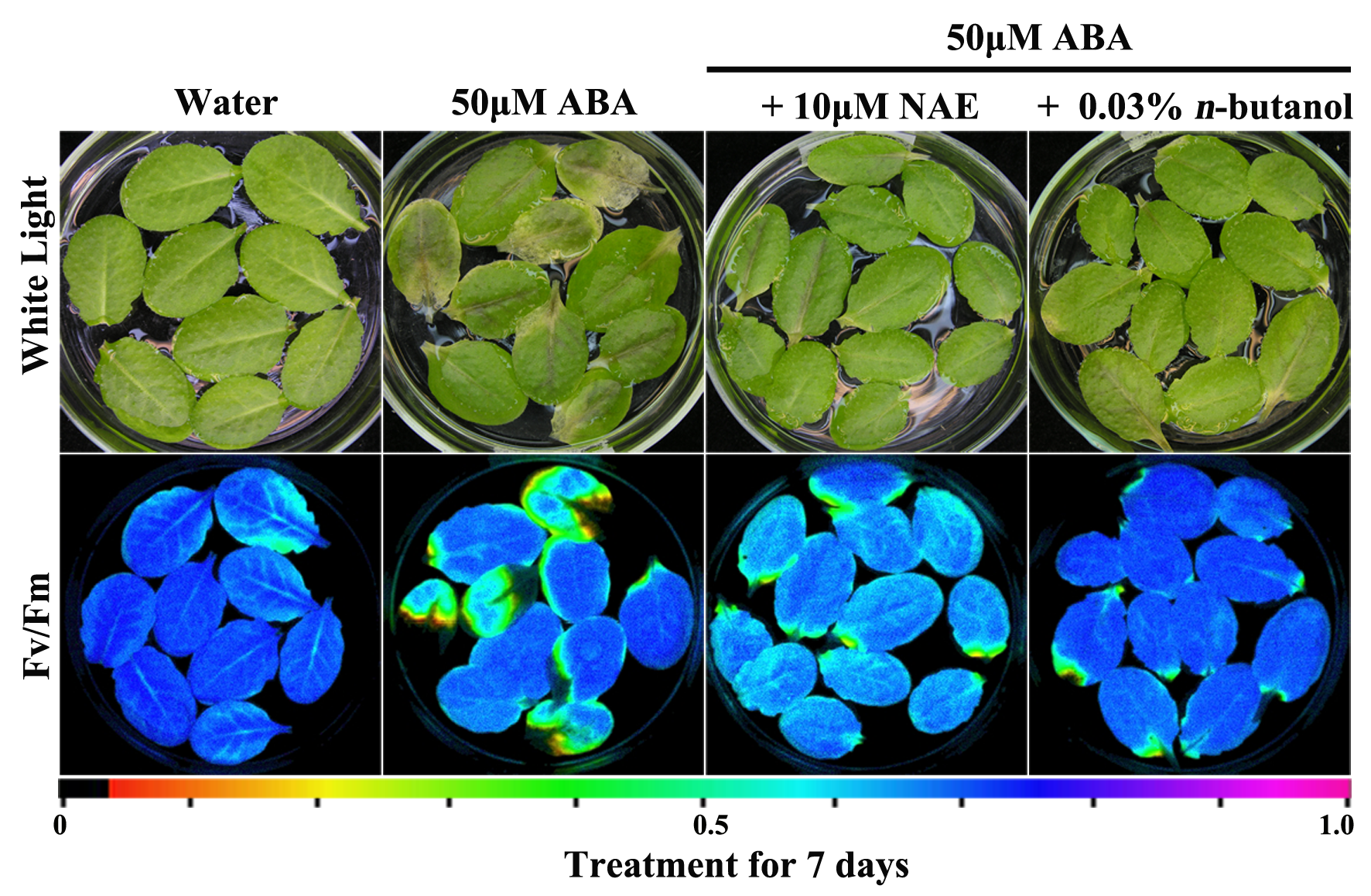

Supplement: Figure S2 — The effects of butanol and NAE on ABA-promoted leaf senescence. Leaves of Arabidopsis WS were detached and floated immediately on water, 50 µM ABA, 50 µM ABA plus 10 µM NAE, or 50 µM ABA plus 0.03% n-butanol for 7 days. The color bar at the bottom indicates Fv/Fm values. (TIF) [file pone.0065687.s002.tif]

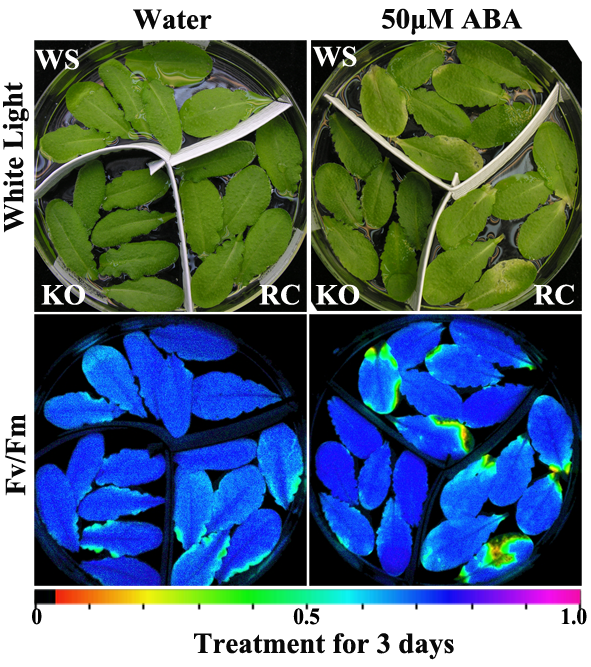

Supplement: Figure S3 — Senescence of detached leaves from WS, PLDδ-KO, and PLDδ-RC plants. Leaves detached from WS, PLDδ-KO, and PLDδ-RC plants were treated with sterile water or 50 µM ABA for 3 days. ABA-promoted senescence was compared among WS, PLDδ-KO, and PLDδ-RC leaves. Yellow coloration (top panel) or low Fv/Fm values for variable fluorescence (bottom panel) indicated the degree of senescence. The color bar at the bottom indicates Fv/Fm values. (TIF) [file pone.0065687.s003.tif]

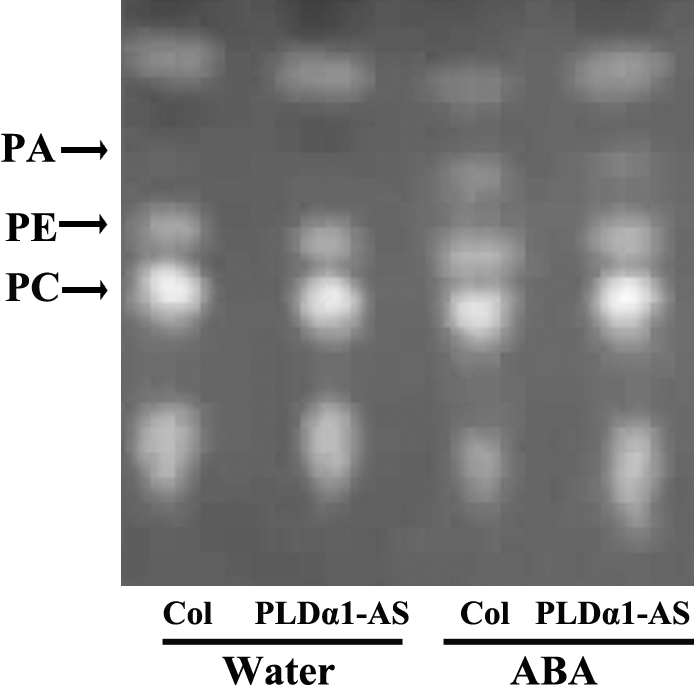

Supplement: Figure S4 — Analysis of PA, PE, and PC in leaves of WT and PLDα1-AS plants during detachment-induced and ABA-promoted senescence, using TLC. Lipid spots were visualized using primuline, and the identification of PA, PC, and PE was verified by comparison of their migration with standards (indicated using arrows). (TIF) [file pone.0065687.s004.tif]
